# Platelets Promote Tumor Growth and Metastasis via Direct Interaction between Aggrus/Podoplanin and CLEC-2

**DOI:** 10.1371/journal.pone.0073609

**Published:** 2013-08-21

**Authors:** Satoshi Takagi, Shigeo Sato, Tomoko Oh-hara, Miho Takami, Sumie Koike, Yuji Mishima, Kiyohiko Hatake, Naoya Fujita

**Affiliations:** 1 Division of Experimental Chemotherapy, Cancer Chemotherapy Center, Japanese Foundation for Cancer Research, Tokyo, Japan; 2 Division of Clinical Chemotherapy, Cancer Chemotherapy Center, Japanese Foundation for Cancer Research, Tokyo, Japan; Vanderbilt University, United States of America

## Abstract

The platelet aggregation-inducing factor Aggrus, also known as podoplanin, is frequently upregulated in several types of tumors and enhances hematogenous metastasis by interacting with and activating the platelet receptor CLEC-2. Thus, Aggrus–CLEC-2 binding could be a therapeutic molecular mechanism for cancer therapy. We generated a new anti-human Aggrus monoclonal antibody, MS-1, that suppressed Aggrus–CLEC-2 binding, Aggrus-induced platelet aggregation, and Aggrus-mediated tumor metastasis. Interestingly, the MS-1 monoclonal antibody attenuated the growth of Aggrus-positive tumors *in vivo*. Moreover, the humanized chimeric MS-1 antibody, ChMS-1, also exhibited strong antitumor activity against Aggrus-positive lung squamous cell carcinoma xenografted into NOD-SCID mice compromising antibody-dependent cellular cytotoxic and complement-dependent cytotoxic activities. Because Aggrus knockdown suppressed platelet-induced proliferation *in vitro* and tumor growth of the lung squamous cell carcinoma *in vivo*, Aggrus may be involved in not only tumor metastasis but also tumor growth by promoting platelet-tumor interaction, platelet activation, and secretion of platelet-derived factors *in vivo*. Our results indicate that molecular target drugs inhibiting specific platelet–tumor interactions can be developed as antitumor drugs that suppress both metastasis and proliferation of tumors such as lung squamous cell carcinoma.

## Introduction

Most circulating tumor cells are rapidly destroyed by shear forces or attacked by the immune system; thus, less than 0.1% of tumor cells survive for 24 h in the bloodstream [1]. Platelets have been reported to play a role in the survival, growth, migration, and EMT of tumor cells in circulation. Several growth factors such as vascular endothelial growth factor, platelet-derived growth factor, and transforming growth factor-β are stored in platelet granules and released on platelet activation. Such platelet-derived factors reportedly induce tumor vascular angiogenesis, growth, and EMT [2–4]. In addition, platelet–tumor interactions are also known to be associated with hematogenous metastasis because antiplatelet agents and thrombocytopenia reportedly reduce the number of experimental tumor metastasis [5–7]. Platelets may facilitate tumor cell survival in circulation by increasing the formation of tumor cell clusters that enhance embolization in the microvasculature and protect the tumor cells from immunological assault or shear stress [8].

Aggrus, also known as podoplanin, T1alpha, or gp36, is a type-I transmembrane sialoglycoprotein that is frequently upregulated in many types of tumors, including squamous cell carcinoma, mesothelioma, Kaposi’s sarcoma, testicular germ cell tumor, and brain tumor [9–12]. Enhanced Aggrus expression in cancer-associated fibroblasts (CAF) has also been reported, and Aggrus expression in CAFs predicted poor prognosis of lung adenocarcinomas [13]. Moreover, Aggrus expression is found in tumor-initiating cells, suggesting the importance of Aggrus in cancer progression [14]. We previously discovered Aggrus as a platelet aggregation-inducing factor expressed in highly metastatic cancers [15]. Aggrus expression accelerates both experimental and spontaneous pulmonary metastasis without affecting tumor growth *in vivo* [7,15]. Aggrus contains three tandem repeats of the conserved motif EDXXVTPG (where X may be any amino acid) in the extracellular domain. We designated the segments as platelet aggregation-stimulating (PLAG) domains because these domains are critical for Aggrus-mediated platelet-aggregating ability [15]. Further research clarified that the PLAG domain is structurally and functionally conserved in primates, carnivores, and rodents [16]. Because forced expression of wild-type Aggrus but not mutant Aggrus lacking platelet-aggregating ability changed non-metastatic CHO cells to metastatic cells [7] and enhanced the rate of arrest in the lung [7,17], the platelet aggregation-inducing activity of Aggrus was directly linked to metastasis. The C-type lectin-like receptor 2 (CLEC-2) expressed on platelets was recently identified as one of the counter receptors of Aggrus [18,19]. Aggrus binding to CLEC-2 transmits platelet-activation signals through Src family kinases, Syk, and phospholipase Cγ2 in platelets [20,21]. CLEC-2–deficient platelets have been reported to respond normally to platelet agonists, such as collagen, ADP, U46619, and PAR-4, which suggests that inhibition of the Aggrus–CLEC-2 interaction may not affect physiological hemostasis [22].

Thus, Aggrus is expected to be a promising therapeutic target for developing antitumor and antimetastatic agents. Although we and another group have previously established many monoclonal antibodies (mAbs) against Aggrus [23–25], the antitumor activities of anti-Aggrus mAbs have not been fully demonstrated. To clarify the therapeutic potential of Aggrus targeting, we generated a new anti-human Aggrus mAb, designated as MS-1. *In vitro* and *in vivo* analysis revealed that MS-1 mAb inhibited Aggrus–CLEC-2 interaction, Aggrus-induced platelet aggregation, and Aggrus-induced pulmonary metastasis. MS-1 mAb administration also exhibited antitumor effects *in vivo* with concurrent spontaneous suppression of pulmonary metastasis of Aggrus–expressing cells. Moreover, humanized chimeric MS-1 antibody suppressed *in vivo* growth of human lung squamous carcinoma PC-10 cells expressing endogenous Aggrus protein on their cell surface. Aggrus knockdown also suppressed the *in vivo* tumor growth and *in vitro* platelet-dependent proliferation of a lung squamous cell carcinoma cell line. These results strongly indicate that platelets are involved in tumor growth and metastasis *in vivo* and that Aggrus is a promising therapeutic target for tumors such as lung squamous cell carcinoma.

## Materials and Methods

### Cell lines

CHO cells were purchased from the American Type Culture Collection (ATCC) and cultured in RPMI 1640 media (Wako, Osaka, Japan) containing 10% FBS (RPMI growth medium, Sigma-Aldrich, St. Louis, MO). PC-10 (Immuno-Biological Laboratories, Gunma, Japan) and A549 (ATCC) cells were cultured in DMEM (Sigma) containing 10% FBS. CHO cells that had been stably transfected with vectors containing none (CHO/mock), human *aggrus* (CHO/Aggrus), and *aggrus* point mutants (CHO/Aggrus-G45A or -D49A) were established in our laboratory and cultured in a medium containing 1 mg/mL of G418 (Life Technologies). PC-10 cells that had been stably expressed with vectors containing none (PC-10/shCont) and human *aggrus*-targeting shRNA (PC-10/shAgg1 and PC-10/shAgg2) were established in our laboratory and cultured in a medium containing 1 µg/mL of puromycin (Life Technologies).

### Plasmid Construction

A human *aggrus* cDNA gene was cloned into pcDNA3 vector (Life Technologies, Carlsbad, CA), as described previously [15]. A human *aggrus* cDNA region encoding P4262 (124–186 b.p.; 42–62 aa) was cloned and connected 18 times repeatedly on a pGEX-6P-3 vector (GE Healthcare, Buckinghamshire, UK). The GST-tagged human ∆N20 *aggrus* cDNA (∆N20) and its point mutants in a pGEX-6P-3 vector were described previously [23]. MISSION shRNA targeting human *aggrus* (TRCN0000061924: shAgg1 and TRCN0000061926: shAgg2) and empty vector (SHC001: shCont) were purchased from Sigma-Aldrich. Targeting sequences of shRNAs were as follows: shAgg1: 5’- CCGGGCTATAAGTCTGGCTTGACAACTCGAGTTGTCAAGCCAGACTTATAGCTTTTTG-3’, shAgg2: 5’- CCGGCAACAACTCAACGGGAACGATCTCGAGATCGTTCCCGTTGAGTTGTTGTTTTTG-3’. ZsGreen expressing vector (pZsGreen-N1) was purchased from TAKARA Bio (Shiga, Japan).

### Hybridoma production and F(ab’)_2_ preparation

The method of hybridoma production has been described previously [23]. A F(ab’)_2_ preparation kit was used according to the manufacturer’s instructions (Thermo, Fisher Scientific, Waltham, MA) to prepare the F(ab’)_2_ of control mouse IgG2a (Sigma) and MS-1 mAb.

### Immunoblot analysis

Cells were lysed in TENSV buffer (50 mM Tris–HCl, pH 7.5, 2 mM EDTA, 100 mM NaCl, 1 mM Na _3_VO_4_, 1% NP-40, 0.1% aprotinin, and 2 mM phenylmethylsulfonyl fluoride) at 4°C with sonication. Sampling buffer (42 mM Tris–HCl, pH 6.8, 10% glycerol, 2.3% SDS, 5% 2-mercaptoethanol, and 0.002% bromophenol blue) was added to each sample, which were subsequently boiled for 5 min and electrophoresed on an SDS-polyacrylamide gel. The proteins were transferred to nitrocellulose transfer membranes (GE Healthcare) and immunoblotted with MS-1 mAb, anti-Aggrus antibodies (clone D2-40; AbD Serotec, Oxford, UK, clone FL-162; Santa Cruz Biotechnology, Santa Cruz, CA), or anti-α-tubulin antibody (clone YL1/2; AbD Serotec). We detected the protein expression using an enhanced chemiluminescence reagent (GE Healthcare) and luminescence image analyzer LAS-3000 mini (Fujifilm, Tokyo, Japan) was performed. GST-tagged human ∆N20-Aggrus (∆N20) and its point mutants were prepared from BL21 *Escherichia Coli* (Life Technologies) and detected by MS-1 mAb or anti-GST antibody (Abcam, Cambridge, UK).

### Immunohistochemistry

Frozen section of xenografted tumors were fixed by paraformaldehyde and treated with peroxidase-blocking solution (DAKO, Glostrup, Denmark). Anti-human Aggrus mAb (clone: D2-40, DAKO) was treated for 45 min at room temperature, followed by incubation with EnVision+ System-HRP labeled polymer anti-mouse (DAKO). Anti-CD41 antibody (clone: MWReg30, GeneTex, Irvine, CA) was treated, followed by incubation with anti-rat IgG HRP detection kit (BD, Franklin Lakes, NJ). Color was developed with ImmPACT DAB (Vector Laboratories, Burlingame, CA). Mayer’s hematoxylin solution (Wako, Osaka, Japan) was used for nuclei counterstain. Scale bar indicated 0.1 µm.

### Enzyme-linked solvent assay

Recombinant (His)_10_-tagged human CLEC-2 was immobilized on plates. After blocking, the plates were further incubated with human IgG Fc-conjugated recombinant human Aggrus protein in the presence of control mouse IgG or MS-1 mAb. The plates were then incubated with peroxidase-conjugated anti-human IgG antibody followed by adding 1-Step Ultra TMB–ELISA reagent (Pierce). The reaction was stopped by adding 2 M sulfuric acid, and the absorbance was measured at 450 nm.

### Cell viability assay

To examine the effects of platelets on PC-10 or Aggrus-knockdown PC-10 cell growth, we established ZsGreen-expressing cell lines and measured fluorescence intensity of ZsGreen as relative cell viability.

### Flow cytometric analysis

Cells were harvested and treated with 1 µg/ml of anti-Aggrus mAbs or control mouse IgG, following incubation with Alexa Fluor 488-conjugated anti-mouse IgG (Life Technologies). In some experiments, cells were incubated with mouse IgG Fc-conjugated human CLEC-2 following incubation with Alexa Fluor 488-conjugated anti-mouse IgG. A Cytomics FC500 flow cytometry system (Beckman Coulter) was used to perform flow cytometric analysis.

### Surface plasmon resonance (SPR) analysis

Biosensor analyses were performed as previously described [23]. In brief, the recombinant human or mouse Aggrus-Fc proteins prepared from conditioned media of mammalian cells (R&D systems, Minneapolis, MN) was covalently attached to a CM5 sensor chip (GE Healthcare). Final levels of immobilization were approximately 200 response units. Five concentrations of MS-1 mAb or ChMS-1 antibody were passed over the chip in a single cycle without regenerating the surface between injections. Sensorgrams were fit by global analysis using the Biacore X100 evaluation software. The equilibrium dissociation constant (*K*_*D*_) was determined from the bivalent analyte model.

### Platelet aggregation assay

Cells (2 × 10^7^ cells/ml) were incubated with 10 µg/ml of MS-1 mAb or control mouse IgG. Murine whole blood was drawn by cardiac puncture from Jcl:ICR mice terminally anesthetized with chloroform and taken with heparin solution. Platelet-rich plasma (PRP) was obtained from supernatant of murine whole blood by centrifugation at 110 ×*g* for 10 min. Washed platelets were prepared from pellets of PRP by centrifugation at 900 ×*g* for 10 min following washing with modified Tyrode’s buffer (20 mM HEPES, 150 mM NaCl, 2.5 mM KCl, 12 mM NaHCO_3_, 1 mg/ml of glucose, 1 mM MgCl_2_, and 1 mg/ml BSA). Washed platelets were resuspended in modified Tyrode buffer and incubated for 30 min at 37°C. Before experiments, 200 µM CaCl_2_ was added to the platelets suspension. Platelet aggregation assay using a platelet aggregometer (MCM HEMA TRACER 313M; SSR Engineering, Kanagawa, Japan) was performed as previously described [23].

### Generation of chimeric humanized antibody

Generation of chimeric antibody has been described previously [23]. In brief, the cDNA of the heavy and light chains of MS-1 mAb were sequenced and analyzed by an online V-Quest software for identification of complementarity-determining region (CDR). The CDR fragments of heavy and light chains were subcloned into heavy or kappa light chain constant region of human IgG1. CHO cells were doubly infected with culture supernatants containing chimeric heavy and kappa light chain-expressing retroviruses. Protein A-Sepharose (Zymed, South San Francisco, CA) was used to purify the chimeric antibody from the supernatant of the infected CHO cells.

### Animals

Female BALB/c-*nu*/*nu* and NOD.CB17-*Prkdc* ^*scid*^/J mice were purchased from Charles River Laboratories Japan, Inc. (Kanagawa, Japan). Jcl:ICR mice were purchased from Clea Japan, Inc. (Tokyo, Japan). All animal procedures were performed according to protocols approved by the Japanese Foundation for Cancer Research Animal Care and Use Committee.

### Metastasis assay

Six-week-old female BALB/c-*nu*/*nu* mice were intravenously administered antibodies into the lateral tail vein the day before cell injection. Cell suspension in Hanks’ Balanced Salt Solution (HBSS, Gibco) was injected intravenously (2.5 × 10^5^ cells/mouse). After 17–20 days, the mice were euthanized and their lungs were stained with saturated picric acid solution. The number of lung surface metastatic foci was counted. Spontaneous metastasis assay was performed as follows. Cell suspension of CHO/Aggrus (1 × 10^6^ cells in 50 µl) in HBSS was subcutaneously inoculated into the backs of eight-week-old female BALB/c-*nu*/*nu* mice. After 35-40 days, the mice were euthanized and the number of lung surface metastatic foci was counted.

### Xenograft models

Cell suspension in HBSS (CHO/Aggrus: 1 × 10^6^, PC-10: 5 × 10^6^, and A549: 1 × 10^6^ cells in 50 µl) was subcutaneously inoculated into the backs of eight-week-old female BALB/c-*nu*/*nu* or NOD.CB17-*Prkdc* ^*scid*^/J mice. Antibodies were intravenously administrated into the lateral tail vein. Tumor volume was calculated by the following formula: volume = W^2^ × L/2, where W is the short diameter and L is the long diameter.

### Statistical analysis

A Mann–Whitney *U* test was performed to determine the statistical significance in the metastasis and xenograft assays. Significant *P*-values are shown as *P < 0.05 or as **P < 0.01. All statistical tests were two-sided. 

## Results

### Establishment of a Novel Anti-human Aggrus mAb, MS-1

To estimate the role of platelets in tumor growth and metastasis, we attempted to establish a new mouse anti-Aggrus mAb because Aggrus has been reportedly associated with pathological platelet aggregation. We generated plasmids that expressed 18-times-repeated human Aggrus-derived antigen (P4262 peptide, AMPGAEDDVVTPGTSEDRYKS; 42-62 aa) and subsequently immunized mice with the new antigen. After hybridoma screening, we obtained several clones that recognized the immunized antigen.

**Figure 1:**
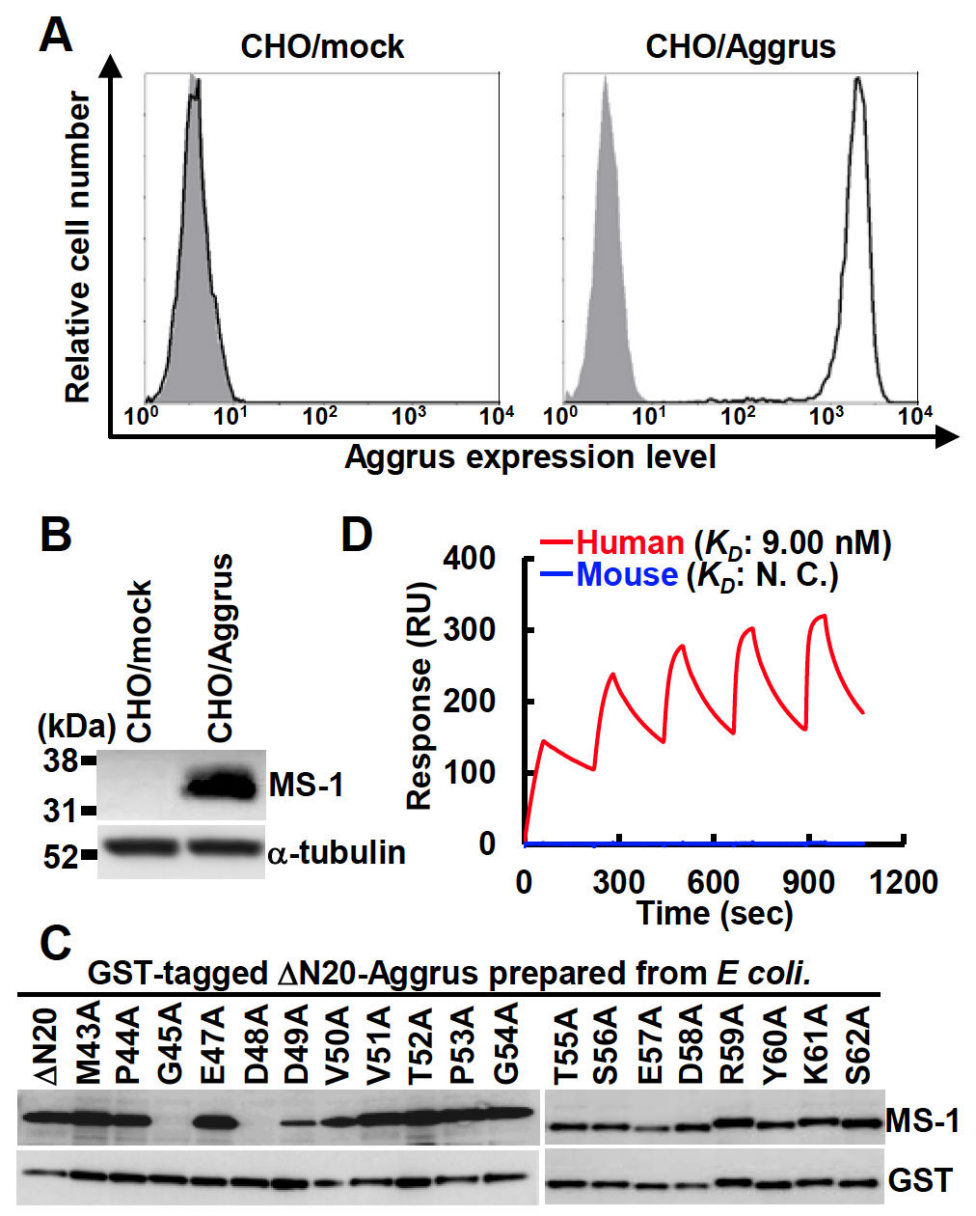
Characterization of the established anti-Aggrus neutralizing mAb MS-1. A, cells were treated with control mouse IgG (gray area) or MS-1 mAb (bold lines). B, cells were lysed and immunoblotted with the indicated antibodies. C, the GST-tagged recombinant human ∆N20-Aggrus protein and its point mutants were expressed in *E. Coli* and immunoblotted with the indicated antibodies. GST, anti-GST antibody. D, interaction between MS-1 mAb and the human Aggrus protein (red line) or the mouse Aggrus protein (blue line) was estimated by SPR analysis. Equilibrium dissociation constants (*K_D_*) of MS-1 mAb on human Aggrus are shown. The *K*_*D*_ of MS-1 mAb on mouse Aggrus could not be calculated (N.C.).

We used a flow cytometer to examine the reactivity of the clones against CHO cells that were transfected with empty vector (CHO/mock) or Aggrus-expressing plasmid (CHO/Aggrus). We established several mAbs and found that MS-1 mAb recognized Aggrus-expressing CHO/Aggrus cells but not CHO/mock cells (Figure 1A). Immunoblot analysis confirmed that MS-1 mAb could bind to the human Aggrus protein (Figure 1B). We used point-mutated Aggrus proteins expressed in *Escherichia coli* to identify the recognition epitopes by alanine scanning. MS-1 mAb could not recognize the Aggrus protein harboring G45A or D48A mutation, indicating that MS-1 mAb recognized the perimeter structure around Gly^45^ and Asp^48^ (Figure 1C). Surface plasmon resonance analysis revealed that MS-1 mAb exhibited high-affinity binding to human Aggrus protein with a *K*_*D*_ of 9.00 nM (Figure 1D, red line) but not to mouse homolog (Figure 1D, blue line), indicating that MS-1 mAb is an anti–human Aggrus-specific mAb with high affinity.

### MS-1 mAb Suppressed Pulmonary Metastasis by Neutralizing the Platelet-Aggregating Ability of Aggrus and by Exhibiting Effector Activity

**Figure 2:**
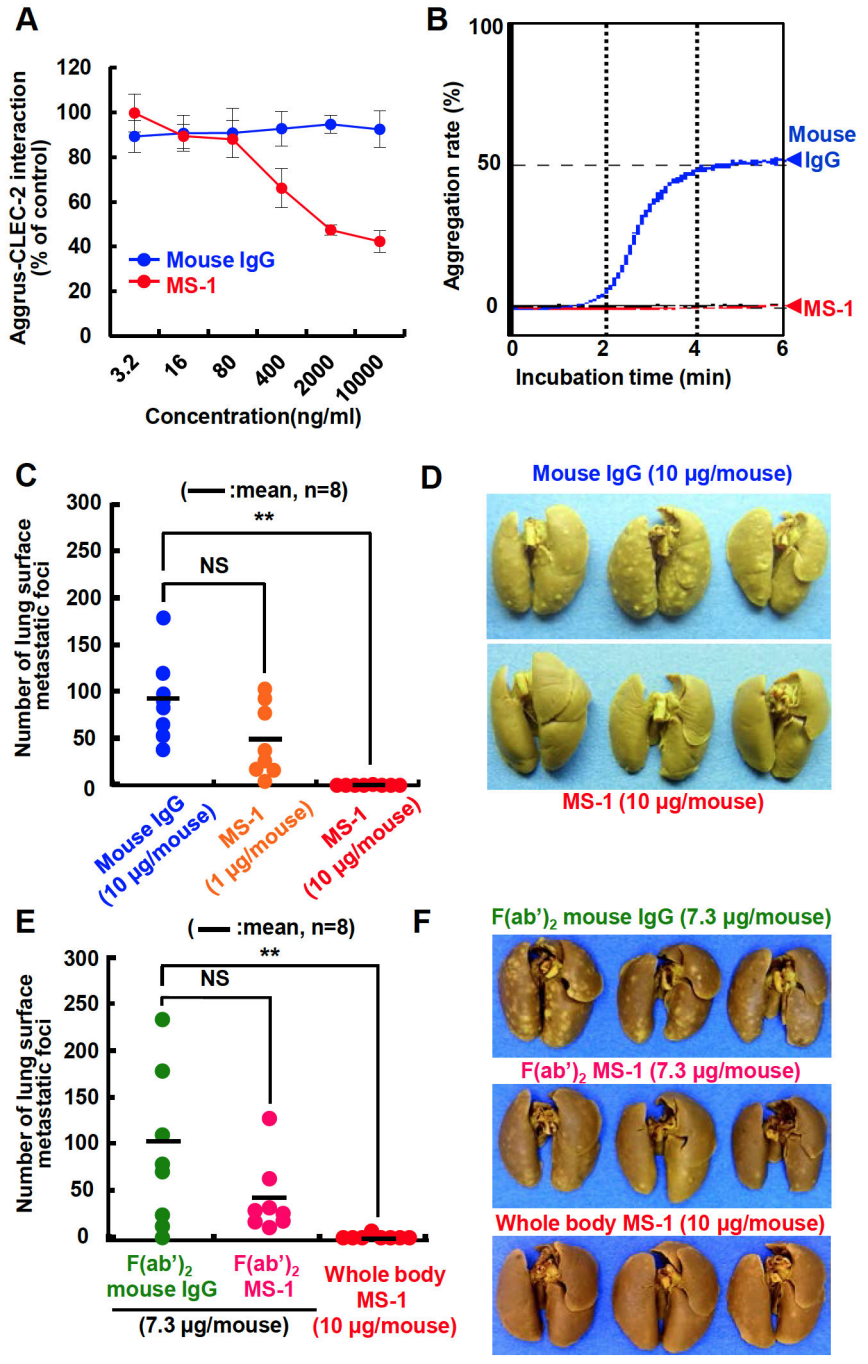
Suppression of Aggrus-induced platelet aggregation and tumor metastasis by MS-1 mAb. A, recombinant CLEC-2 protein was immobilized on an ELISA plate and then incubated with the human IgG Fc-tagged recombinant human Aggrus protein in the presence of the indicated concentrations of control mouse IgG (Mouse IgG) or MS-1 mAb. The value of PBS-treated control was normalized to 100%. Data are means ± SDs of triplicate determinations. B, CHO/Aggrus cells were incubated with 10 µg/mL of antibodies, followed by incubation with mouse PRP. Light transmittance of samples was measured as the aggregation rate. C to F, BALB/c-*nu/nu* mice were intravenously injected with the indicated concentrations of antibodies (C and D) or F(ab’)_2_ fragments (E and F). CHO/Aggrus cells were intravenously inoculated on the following day of antibody administration. After 20 days of tumor inoculation, lung surface metastatic foci were counted. Numbers of metastatic foci in each mouse were shown. Bars, mean (n=8). NS, not significant. **P < 0.01 by the Mann–Whitney *U* test (C and E). Representative pictures of the lungs and lung surface metastatic foci are shown (D and F).

To estimate the function of the established MS-1 mAb, we first performed an ELISA-based competition assay to clarify the inhibitory effect of the antibody on Aggrus–CLEC-2 binding. As shown in Figure 2A, MS-1 mAb inhibited the Aggrus–CLEC-2 interaction in a concentration-dependent manner. Because the Aggrus–CLEC-2 interaction is critical for Aggrus-induced platelet aggregation and tumor metastasis [9,18], we investigated its effect on Aggrus-induced platelet aggregation *in vitro* and pulmonary metastasis *in vivo*. As expected, the MS-1 mAb suppressed Aggrus-induced platelet aggregation (Figure 2B) and pulmonary metastasis (Figure 2C and 2D). To eliminate the effector activity mediated by the Fc region of MS-1, we generated an F(ab’)_2_ fragment of MS-1 mAb [F(ab’)_2_ MS-1] using pepsin digestion. Administration of the purified F(ab’)_2_ fragment decreased the number of metastatic foci of CHO/Aggrus cells by half (Figure 2E and 2F). These results indicated that MS-1 mAb suppressed Aggrus-induced hematogenous metastasis not only by inhibiting Aggrus–CLEC-2 binding but also by exhibiting effector activity.

### Binding to the Specific Epitope Is Essential for Exhibiting Antimetastatic Activity

**Figure 3:**
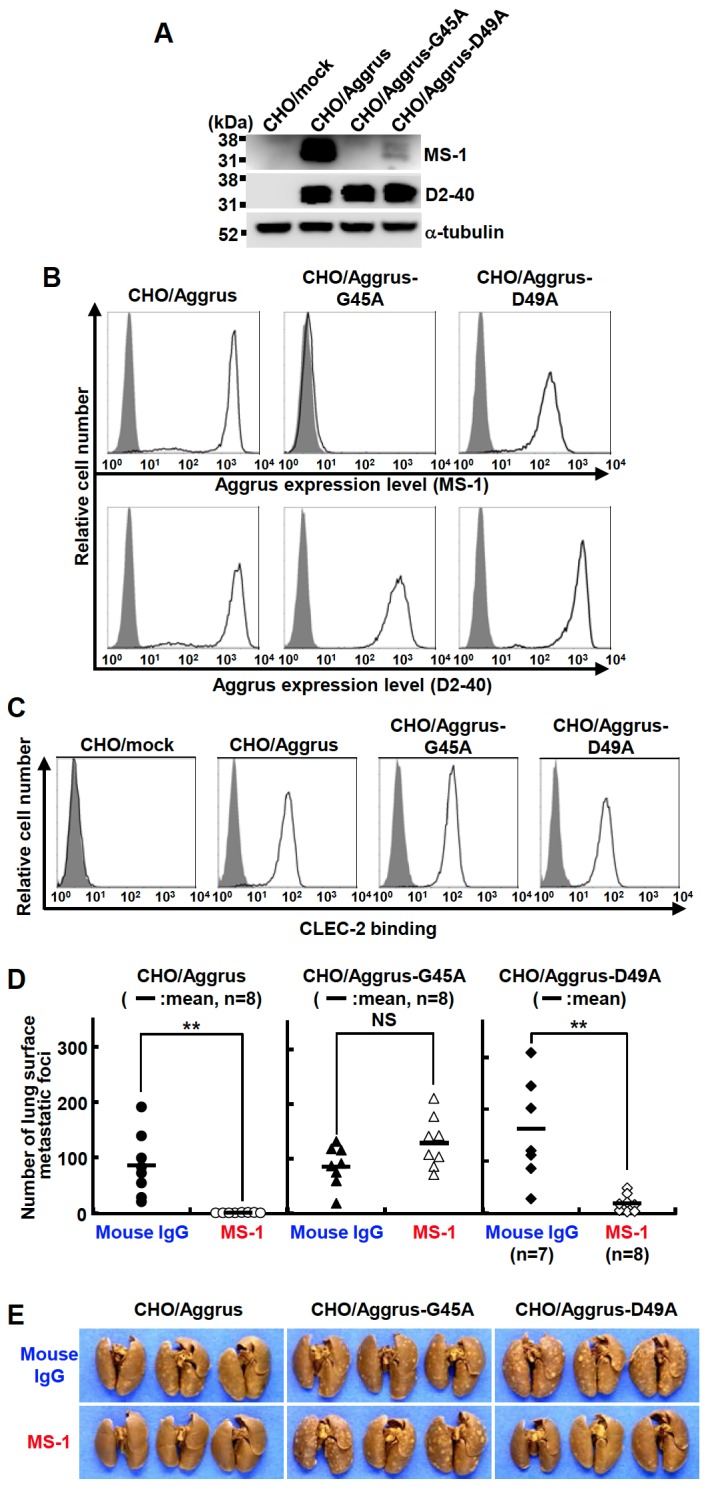
Identification of recognition epitope of MS-1 mAb on human Aggrus. A, cells were lysed and immunoblotted with the indicated antibodies. B, CHO cells that had been transfected with WT-, G45A- or D49A-Aggrus expression plasmids (CHO/Aggrus, CHO/Aggrus-G45A or CHO/Aggrus-D49A, respectively) were treated with control mouse IgG (0.1 µg/ml, gray area) or MS-1 mAb (0.1 µg/ml, bold lines in upper panels). In some experiments, cells were treated with an anti-human Aggrus mAb D2-40 antibody (0.1 µg/ml, bold lines in lower panels). After incubation with the Alexa Fluor 488-conjugated secondly antibody, Aggrus expression was detected by flow cytometry. C, CHO/mock, CHO/Aggrus, CHO/Aggrus-G45A, and CHO/Aggrus-D49A cells were treated with control mouse IgG (0.1 µg/ml, gray area) or mouse IgG Fc-conjugated recombinant human CLEC-2 protein (4 µg/ml, bold lines). After incubation with the Alexa Fluor 488-conjugated secondly antibody, CLEC-2 binding to Aggrus-expressing cells was confirmed by flow cytometry. D and E, BALB/c-*nu/nu* mice were intravenously injected with control mouse IgG (Mouse IgG) or MS-1 mAb (3 µg/mouse). After 24 h, cells (2.5 × 10^5^ cells/mouse) were intravenously inoculated into mice. After 20 days of tumor inoculation, lung surface metastatic foci were counted. The number of metastatic foci are shown (D). Bars, mean (n = 7 or 8). NS, not significant. **P < 0.01 by the Mann–Whitney *U* test. Representative pictures of the lungs and lung surface metastatic foci are shown (E).

MS-1 mAb recognized the perimeter structure around Gly^45^ and Asp^48^ because MS-1 mAb could not recognize G45A- or D48A-Aggrus protein expressed in *E. Coli* (Figure 1C). In addition, the reactivity of MS-1 mAb against D49A-Aggrus seemed to be weaker than that against wild-type ∆N20-Aggrus (Figure 1C). To confirm the results in mammalian cells, we generated CHO cells overexpressing wild-type (CHO/Aggrus), G45A (CHO/Aggrus-G45A), or D49A (CHO/Aggrus-D49A) Aggrus proteins. The total expression level of wild-type and point-mutated Aggrus in each transfectant was confirmed using an anti–human Aggrus mAb D2-40 that recognized a different epitope (Figure 3A and 3B). The reactivity of MS-1 mAb against CHO/Aggrus-G45A was abolished, and the reactivity against CHO/Aggrus-D49A cells seemed to be attenuated (Figure 3A and 3B). Substitution of Gly^45^ and Asp^49^ to Ala did not affect their binding to CLEC-2 (Figure 3C). Consistent with these results, both CHO/Aggrus-G45A and CHO/Aggrus-D49A cells formed many metastatic foci in the lung (Figure 3D and 3E). Intravenous administration of MS-1 mAb a day before CHO/Aggrus inoculation completely prevented lung metastasis of the cells. In contrast, pulmonary metastasis of CHO/Aggrus-G45A cells could not be suppressed. CHO/Aggrus-D49A-mediated metastasis was partially inhibited by administration of MS-1 mAb (Figure 3D and 3E). These results indicated that recognition and inhibition of Aggrus function were required to exhibit the antimetastatic ability of MS-1 mAb.

### MS-1 mAb Suppressed Spontaneous Pulmonary Metastasis and Exhibited Antitumor Activity In Vivo

**Figure 4:**
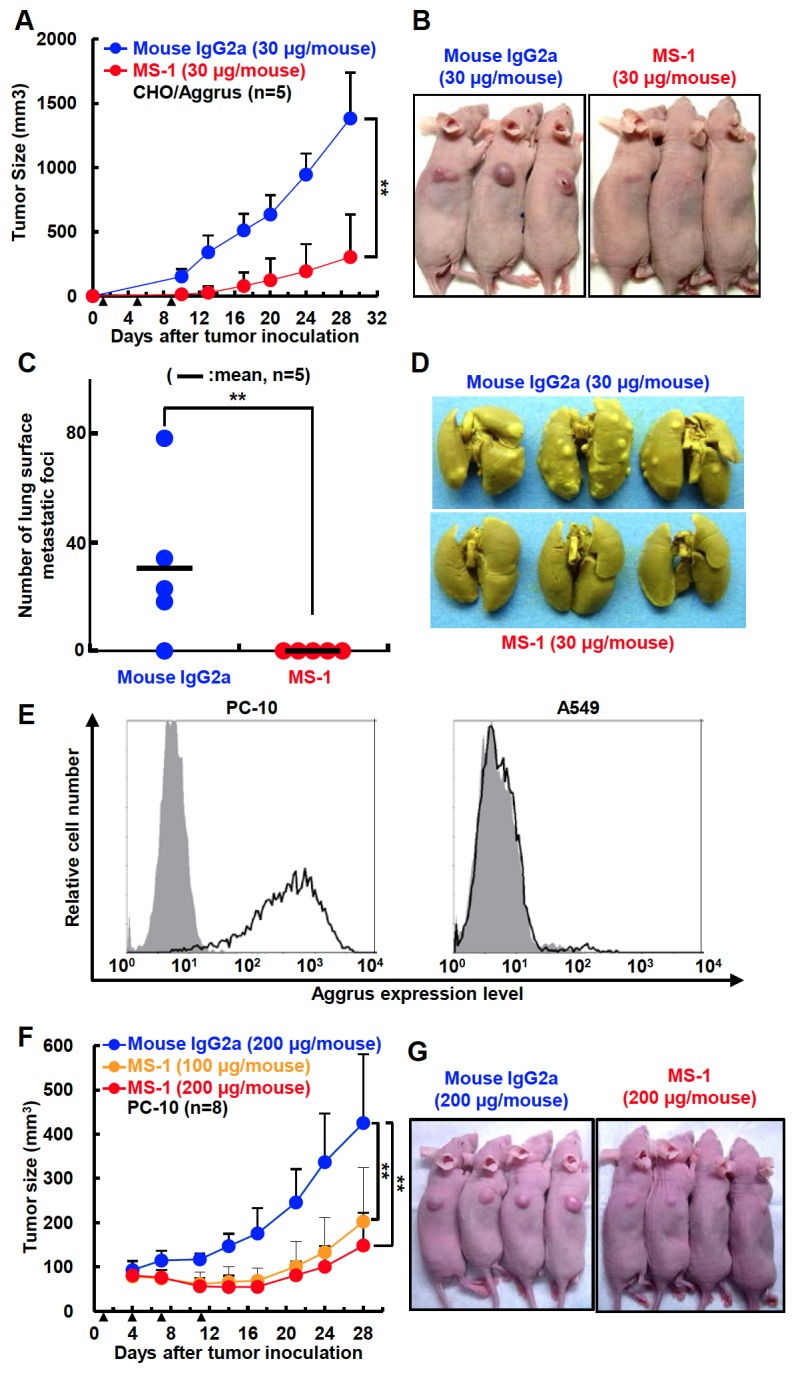
MS-1 mAb suppressed spontaneous pulmonary metastasis and tumor growth *in vivo*. A to D, CHO/Aggrus cells were xenografted in BALB/c-*nu/nu* mice. Antibodies (30 µg/mouse) were intravenously injected on the following day of tumor inoculation and repeated two more times every fourth day (arrow heads). Tumor volume was calculated as described in Materials and Methods. All data are shown as means ± SDs (n=5). **P < 0.01 by the Mann–Whitney *U* test (A). Representative pictures of CHO/Aggrus tumor-bearing mice on day 18 are shown (B). After 29 days of tumor inoculation, spontaneously pulmonary metastatic foci were counted. The numbers of metastatic foci are shown. Bars, mean (n = 5). **P < 0.01 by the Mann–Whitney *U* test (C). Representative pictures of the lungs and lung surface metastatic foci are shown (D). E, a lung squamous cell line PC-10 (left) and a lung adenocarcinoma cell line A549 (right) cells were treated with control mouse IgG (gray area) or MS-1 mAb (bold lines). Aggrus expression was detected by flow cytometry. F and G, PC-10 cells were xenografted into BALB/c-*nu/nu* mice. Antibodies were intravenously injected on 1, 4, 7, and 11 days after tumor inoculation (arrow heads). Tumor volume was calculated as described in Materials and Methods. All data are shown as means ± SDs (n=8). **P < 0.01 by the Mann–Whitney *U* test (F). Representative pictures of the PC-10 tumor-bearing mice on day 18 are shown (G).

We subsequently examined the effects of MS-1 mAb on spontaneous pulmonary metastasis of CHO/Aggrus cells. The days after subcutaneous inoculation of CHO/Aggrus cells in nude mice, we treated the mice with MS-1 mAb twice a week to prevent formation of micro metastasis. Surprisingly, administration of MS-1 mAb suppressed the growth of CHO/Aggrus tumors *in vivo* (Figure 4A and 4B). Under these conditions, MS-1 mAb almost completely suppressed spontaneous metastasis of CHO/Aggrus cells (Figure 4C and 4D). These results indicated that MS-1 mAb possessed antitumor activity and antimetastatic activity. Because Aggrus has been reported to be expressed in many types of cancers [9–14], we estimated the *in vivo* efficacy of MS-1 mAb on tumors endogenously expressing Aggrus on their cell surface. Aggrus expression is reportedly upregulated in lung squamous cell carcinoma but not in lung adenocarcinoma [11–13]. We screened Aggrus expression in lung cancer cell lines and found that a lung squamous cell carcinoma cell line, PC-10, endogenously expressed Aggrus on the cell surfaces (Figure 4E). As shown in Figure 4F and 4G, MS-1 mAb suppressed the growth of PC-10 tumors xenografted into nude mice in a concentration-dependent manner (Figure 4F and 4G). These results suggested that MS-1 mAb exhibits antitumor activity against Aggrus-expressing human tumors.

### Involvement of Aggrus-CLEC-2 Binding in Platelet-mediated Tumor Growth In Vivo

**Figure 5:**
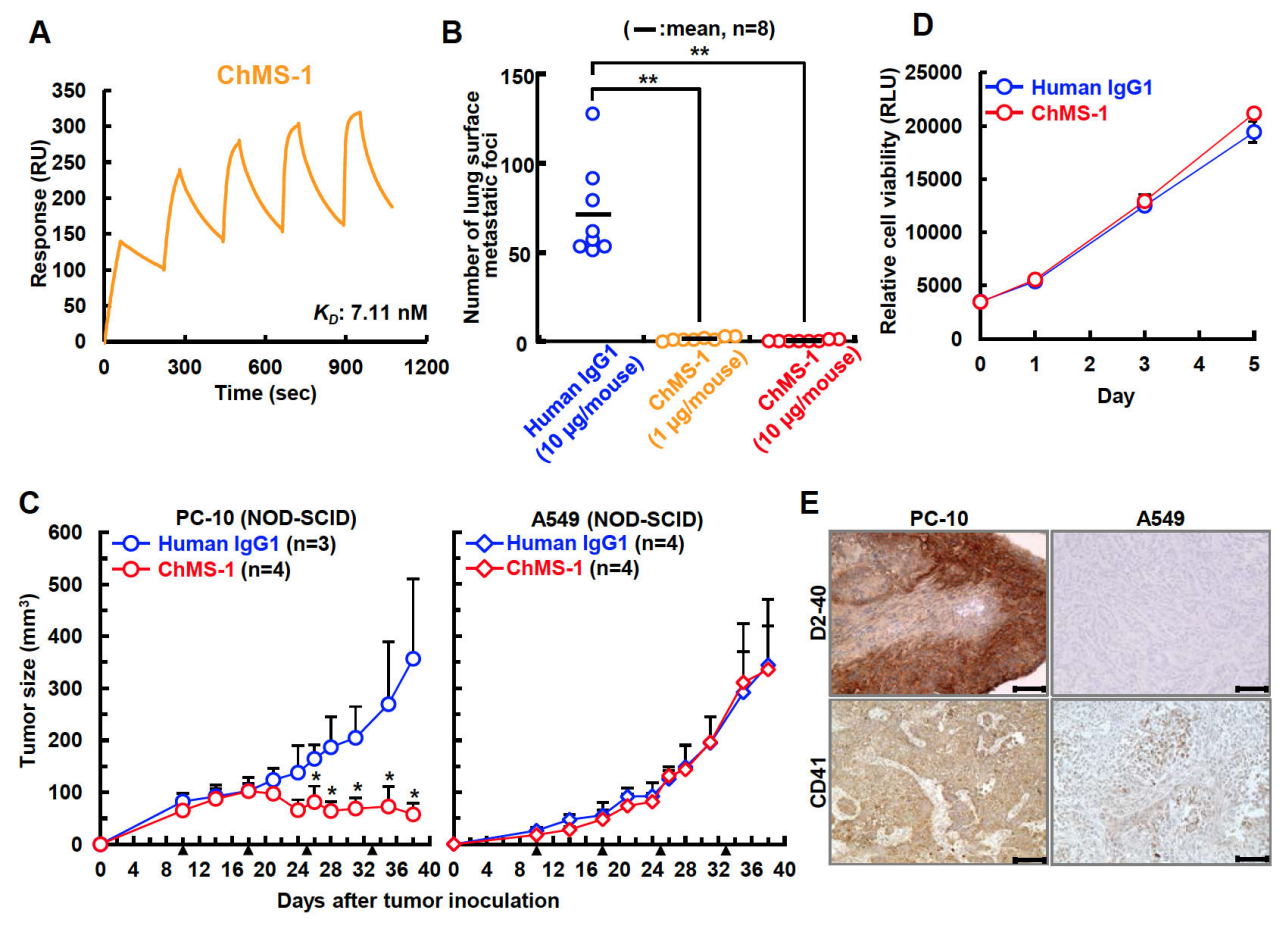
Humanized chimeric ChMS-1 antibody suppressed the *in vivo* growth of Aggrus-expressing lung squamous cell carcinoma PC-10 cells. A, interaction between the human Aggrus protein prepared from mammalian cells (R&D systems) and ChMS-1 antibody was estimated by SPR analysis. *K*_*D*_ of ChMS-1 antibody on human Aggrus is shown. B, antimetastatic activity of ChMS-1 antibody. BALB/c-*nu/nu* mice were intravenously injected with the indicated concentrations of antibodies. After 24 h, CHO/Aggrus cells were intravenously inoculated. After 20 days of tumor inoculation, lung surface metastatic foci were counted. The number of metastatic foci are shown. Bars, mean (n=8). **P < 0.01 by the Mann–Whitney *U* test. C, antitumor activity of ChMS-1 antibody in NOD-SCID mice. PC-10 (left) and A549 (right) cells were subcutaneously inoculated into the backs of eight-week-old female NOD.CB17-*Prkdc* ^*scid*^/J mice. After 10 days of tumor inoculation, antibodies were intravenously injected into the lateral tail vein and repeated 3 more times once a week (100 µg/mouse, arrow heads). Tumor volume was calculated as described in Materials and Methods. All data are shown as means ± SD. *P < 0.05 by the Mann–Whitney *U* test. D, PC-10 cells were cultured in the presence of control human IgG1 or ChMS-1 (10 µg/ml). Cell viability was measured by adding the CellTiter-Glo assay reagent. Briefly, a total of 1,500 cells were seeded in 96-well plates in triplet. On the following day, cells were treated with or without antibodies and incubated for another 24, 72, or 120 hours. Cell viability was determined by adding the CellTiter-Glo assay reagent (Promega) for 10 minutes and luminescence was measured using a Centro LB 960 luminometer (Berthold Technologies). E, representative image of infiltrated platelets (stained by anti-CD41 antibody, lower panels) and Aggrus expression in tumors (stained by D2-40, upper panels). PC-10 and A549 tumors were excised after 40 days of tumor inoculation. Bar, 0.1 µm. Magnification, x20.

Because the effectiveness of MS-1 mAb in Aggrus-targeting therapy was shown in the mouse model, we generated a humanized chimeric MS-1 antibody (ChMS-1). We cloned the complementarity-determining region of MS-1 mAb and ligated it into the constant region of human IgG1. SPR analysis showed that the generated ChMS-1 antibody specifically bound to human Aggrus with affinity similar to that of MS-1 mAb (Figure 5A, K_*D*_ = 7.11 nM). Similar to the mouse MS-1 mAb, the ChMS-1 antibody prevented the experimental metastasis of CHO/Aggrus cells *in vivo* (Figure 5B). To determine the potential contribution of the platelet-neutralizing activity on ChMS-1-mediated antitumor activity, we inoculated Aggrus-positive PC-10 cells and Aggrus-negative A549 cells (Figure 4E) into NOD-SCID mice largely lacking functional NK cells, monocytes, and complement. Interestingly, administration of ChMS-1 antibody significantly suppressed the growth of xenografted PC-10 tumors but not the growth of A549 tumors (Figure 5C). We also confirmed that ChMS-1 mAb did not exhibit cytotoxic effects against PC-10 cells in the absence of mononuclear cells or complement *in vitro* (Figure 5D). These results indicated that the Aggrus–CLEC-2 binding inhibitory activity of ChMS-1 mAb may contribute to the growth retardation of xenografted PC-10 tumors. Therefore, we stained the frozen sections of PC-10 and A549 tumors with antibody recognizing a platelet marker CD41 protein. Surprisingly, several platelets were infiltrated around the PC-10 tumors but not around the A549 tumors (Figure 5E). Platelets are well known to contain several cytokines and growth factors and to secrete them after stimulation. Most are reported to enhance the growth and motility of tumors [2–4]. The infiltration of platelets into tumors and retardation of tumor growth caused by inhibition of Aggrus–CLEC-2 binding suggested that platelet-secreted factors may be associated with the growth of xenografted PC-10 tumors.

### The Importance of Aggrus Expression in In Vivo Growth of PC-10 Cells

**Figure 6:**
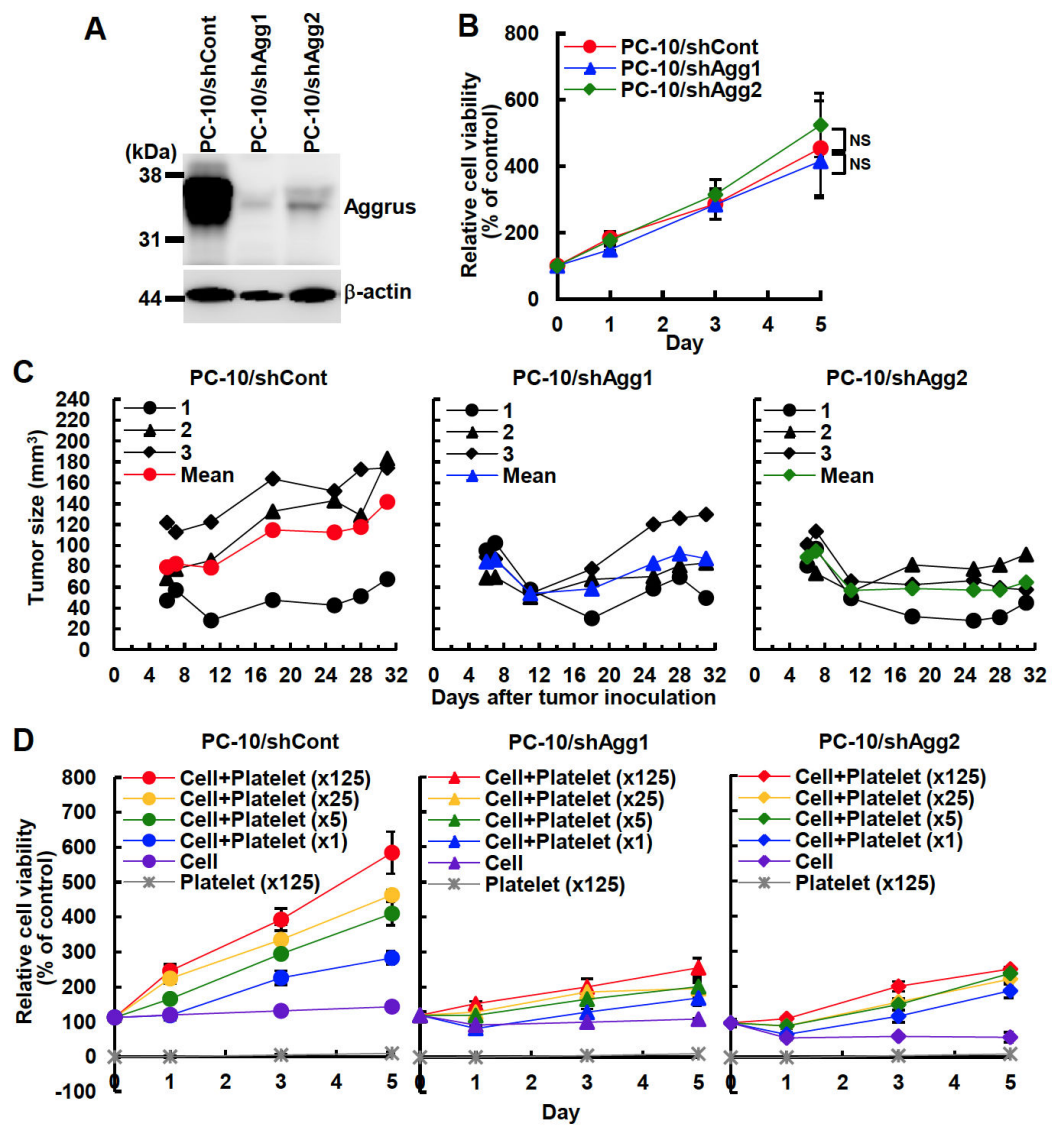
Involvement of Aggrus-platelet interaction in *in vivo* tumor growth. A, establishment of Aggrus-knockdowned PC-10 cells. Aggrus expression of PC-10 transfectants was confirmed by immunoblotting with the indicated antibody. B, *in vitro* growth of PC-10 transfectants that have been stably transfected with ZsGreen-expressing plasmid. Cells were cultured in medium containing 10% FBS for the indicated times. Relative cell viability was calculated as measuring the fluorescence of ZsGreen. C, *in vivo* growth of PC-10 transfectants. PC-10 cells were subcutaneously inoculated into the backs of eight-week-old female BALB/c-*nu/nu* mice (n=3). Tumor volume was calculated as described in Materials and Methods. D, the effects of platelets on PC-10 cell growth *in vitro*. PC-10 transfectants that have been stably transfected with ZsGreen-expressing plasmid were co-cultured with washed platelets in medium containing 0.5% FBS. Relative cell viability was calculated as measuring the fluorescence of ZsGreen.

To confirm the role of Aggrus and Aggrus-platelet interaction on the growth of PC-10 cells *in vivo*, we generated Aggrus-knockdown PC-10 cells (Figure 6A) and compared their proliferation *in vitro* and *in vivo*. Although Aggrus knockdown did not affect the growth of PC-10 cells *in vitro* (Figure 6B), and drastic retardation of *in vivo* tumor growth was observed in both Aggrus-knockdown PC-10 cells (PC-10/shAgg1 and PC-10/shAgg2, Figure 6C). To confirm the role of platelets, we cultured PC-10 transfectants with washed platelets *in vitro* under low serum conditions. As shown in Figure 6D, addition of platelets greatly enhanced the proliferation of control PC-10 cells (PC-10/shCont) in a concentration-dependent manner (Figure 6D). In contrast, the growth of PC-10/shAgg1 and PC-10/shAgg2 cells was barely induced by co-culture with platelets (Figure 6D). These results suggest that platelets may promote PC-10 cell growth only when they were activated by interacting with Aggrus protein on tumor cell surfaces. The results strongly suggest that Aggrus is not only associated with tumor metastasis but also with tumor growth by promoting interaction with platelets.

## Discussion

Platelets and platelet aggregation are associated with cancer progression. Platelet–tumor interaction is considered to contribute to the progression of tumor malignancy by the following three modes of action: (i) tumor cells coated by platelets in the vasculature become bulky and adhesive, and these properties increase the rate of tumor embolization in the microvasculature; (ii) platelets protect tumor cells from shear stress and immunological assault in the bloodstream by coating their cell surfaces; and (iii) growth factors secreted from granules of activated platelets enhance the growth and motility of tumors and tumor vasculature. In fact, the common types of anticoagulants such as heparin and warfarin have been reported to suppress tumor growth, metastasis, and angiogenesis in mouse models both *in vitro* and *in vivo* [26,27]. Moreover, some clinical trials have clarified the efficacy of antiplatelet and anticoagulant drugs for tumor therapy. Administration of low-molecular-weight heparin prolonged the overall survival of patients with advanced malignancy [28,29]. Long-term administration of aspirin decreased the risk of colorectal cancer and recurrence of adenomatous polyps [30]. However, the most important issue related to administration of antiplatelet and anticoagulant drugs for cancer therapy is the increased bleeding risk, and it is not appropriate to administrate these drugs to cancer patients who often require platelet transfusions because of chemotherapeutic drug toxicity-induced thrombocytopenia. Furthermore, administration of antiplatelet and anticoagulant drugs for patient who are planning to undergo or who have just undergone surgery also does not seem to be appropriate. Therefore, a novel and low-bleeding approach is necessary for suppressing specific platelet–tumor interactions.

Platelet aggregation-inducing factor Aggrus can induce platelet aggregation by interacting with its counterpart CLEC-2 expressed on platelet surfaces. The fact that CLEC-2^-/-^ platelets normally respond to several platelet agonists except for rhodocytin, a snake toxin that interacts and activates CLEC-2, indicates that inhibition of Aggrus–CLEC-2 interaction should not interfere with physiological hemostasis [22]. Hence, our generated MS-1 or ChMS-1 mAb that can neutralize Aggrus–CLEC-2 binding is expected to be safe and useful as an antitumor and antimetastatic agent.

Effector activities, including ADCC and CDC, are important mechanisms of action for therapeutic antibodies approved for clinical usage (i.e., trastuzumab and rituximab); however, cellular cytotoxicity would also be extended to the normal tissues that express antigens of therapeutic antibodies. Because Aggrus expression has also been reported in normal tissues, including lymphatic vessels, kidney podocytes, mesothelium, and alveolar epithelium, antibodies that exhibit potent effector activity may not be suitable as therapeutic agents for Aggrus-positive cancers. In this study, we found that our generated humanized chimeric antibody ChMS-1 could inhibit tumor growth of lung squamous cell carcinoma xenografted in NOD-SCID mice largely lacking functional NK cells, monocytes, and complement (Figure 4F and 4G). Thus, effector activities, including ADCC and CDC, would not be required for tumor growth inhibition by anti-Aggrus neutralizing antibodies. Therefore, a molecular target drug that inhibits specific tumor-platelet interactions would be useful as a new type of antitumor drug.

No comprehensive genome analysis of lung squamous carcinomas has identified the region of somatic copy number amplification of PDGFRA and FGFR1 [31,32]. Moreover, the Cancer Genome Atlas Research Network that demonstrated comprehensive genomic characterization of lung squamous cell carcinomas has reported FGFRs as one of the potential therapeutic targets against lung squamous cell carcinoma [33]. Platelets are enriched in a number of growth factors such as PDGFs, FGF-2, and EGF [8,34]. These factors are released after platelet activation, and the secreted factors may contribute to tumor growth and tumor angiogenesis. In fact, we clarified that interaction with and activation of platelets induced the growth of PC-10 cells through Aggrus–CLEC-2-mediated platelet aggregation. Thus, ChMS-1 antibody would suppress the growth of lung squamous cell carcinoma *in vivo* by disrupting the platelet aggregation that leads to secretion of growth factors from platelets.

Finally, Aggrus has been reported to be upregulated in squamous cell carcinomas of the lung, esophagus, oral, head and neck, and skin. It is important to note that approximately 400,000 people die from lung squamous cell carcinoma each year worldwide; development of molecular targeting drugs for lung squamous cell carcinoma is therefore critical [33]. Moreover, the rate of hematogenous metastasis is high in lung squamous cell carcinomas. Because 60% of lung squamous cell carcinomas express Aggrus and because this expression enhanced the risk of metastasis, ChMS-1 antibody may be used as a therapeutic drug for the treatment of lung squamous cell carcinomas. Furthermore, Aggrus-Fc protein produced in the skin of transgenic mice has been reported to induce disseminated intravascular coagulation, and 15% of the mice died at 3-6 weeks of age [35]. Therefore, ChMS-1 antibody may also be used as a therapeutic drug to improve the quality of life or survival rate of patients with Aggrus-positive tumors or Aggrus-induced thrombosis.
